# Expression and prognostic value of carbonic anhydrase IX (CA-IX) in bladder urothelial carcinoma

**DOI:** 10.1186/s12894-022-01074-9

**Published:** 2022-08-03

**Authors:** An-Ping Xiang, Xiao-Nong Chen, Peng-Fei Xu, Si-Hai Shao, Yue-Fan Shen

**Affiliations:** Department of Urology, The First People’s Hospital of Huzhou, #158, Square Road, Huzhou, 313000 China

**Keywords:** Bladder urothelial carcinoma, Carbonic anhydrase IX, Recurrence

## Abstract

**Objective:**

To investigate the expression intensity of carbonic anhydrase IX (CA-IX) in bladder urothelial carcinoma and its predictive value for the recurrence after transurethral resection of bladder tumor.

**Methods:**

A retrospective analysis was made of 194 specimens who underwent transurethral resection of bladder tumors in our hospital from January 2014 to January 2016 and completed follow-up. The expression intensity of CA-IX and the clinical data of the patients were analyzed, and the subjects were divided into positive group and negative group according to the expression intensity of CA-IX. The age, gender, T stage, degree of differentiation, tumor number, tumor diameter, recurrence of each group was analyzed. Logistic univariate and multivariate analysis was used successively to find independent influencing factors for predicting the recurrence of bladder urothelial carcinoma after resection. The Kaplan–Meier survival curve was drawn according to the relationship between CA-IX expression intensity and postoperative recurrence.

**Results:**

The positive expression rates of CA-IX in bladder urothelial carcinomas were 68.1% (132/194). The positive expression of CA-IX had no statistical significance with age, gender and tumor diameter (*P* > 0.05), while the positive expression of CA-IX had statistical significance with tumor T stage, tumor differentiation, tumor number and recurrence (*P* < 0.05); Logistic regression analysis showed that clinical T stage, tumor differentiation, tumor number, and CA-IX expression intensities were independent risk factors for predicting recurrence of bladder urothelial carcinoma after resection (*P* < 0.05); There were 59 cases of recurrence in the positive expression of CA-IX group, with a recurrence rate of 44.69% (59/132), and 17 cases of recurrence in the negative expression group, with a recurrence rate of 27.41% (17/62). The mean recurrence time of CA-IX positive group was 29.93 ± 9.86 (months), and the mean recurrence time of CA-IX negative group was 34.02 ± 12.44 (months). The Kaplan–Meier survival curve showed that the recurrence rate and recurrence time of patients with positive expression of CA-IX in bladder urothelial carcinomas were significantly higher than those of patients with negative expression of CA-IX.

**Conclusion:**

CA-IX is highly expressed in bladder urothelial carcinoma, is a good tumor marker, and can be used as a good indicator for predicting the recurrence of bladder urothelial carcinoma after transurethral resection of bladder tumor.

## Introduction

Bladder urothelial carcinoma is a common disease worldwide, and its mortality rate ranks 13th [1]. At present, imaging examination and cystoscopy are the principal clinical diagnosis methods, and surgery is the clinical treatment method. Early diagnosis can significantly improve the treatment effect and cure rate [2]. Therefore, it is very important to find tumor markers for bladder urothelial carcinoma, because early detection of tumors can reduce the economic burden [3].

Bladder cancer can be divided into non-muscle invasive bladder cancer (NMIBC) and muscle invasive bladder cancer (MIBC), Non-muscle invasive bladder cancer occurs in approximately 70 to 80 percent of patients with primary diagnosis [4]. Transurethral resection of bladder tumor (TURBT) is the main treatment method for non-muscle-invasive bladder cancer, while radical cystectomy is required for muscle-invasive bladder cancer [5]. Bladder cancer is characterized by multiple lesions and easy recurrence. [6], A lot of numbers have the recurrence after TURBT or radical cystectomy and were forced to undergo reoperation or radiotherapy and chemotherapy [7], which added pain to the patients and caused Potential doctor-patient conflict, and the tumor-specific survival rate of patients is reduced. Pathological staging and grading of postoperative specimens are the main key indicators for predicting prognosis, but this traditional method has certain limitations. Finding quick and convenient tumor markers is a hot point in clinical work, which can guide doctors to intervene the patients with poor prognosis in advance and reduce the recurrence and progression of tumor [8].

It CA-IX is a member of the CA family [9], which is expressed on the cell membrane and participates in the reaction of carbon dioxide and bicarbonate in human cells [10]. The main role is to regulate the acid–base stability of cells under hypoxic environment, so that cells can tolerate hypoxia. Current studies have found that CA-IX is a new type of tumor antigen, which is involved in the formation and invasion of tumors, so that tumors still have strong viability under hypoxic conditions [11]. Hypoxia and low pH environment can promote the invasion and metastasis of cancer cells. A number of studies have revealed that CA-IX can be highly expressed in a variety of malignant tumors. It has been reported in the literature that the expression of CA-IX is related to the migration and invasion of renal cancer, lung cancer, and breast cancer [12]. CA-IX is expected to be a good tumor marker for predicting tumor recurrence [13, 14].

## Objects and methods

### Objects

Inclusion criteria: From January 2014 to January 2016, the patients were diagnosed with non-muscle invasive bladder urothelial carcinoma in the Department of Pathology of the First People's Hospital of Huzhou City, Zhejiang Province, and underwent transurethral resection of bladder tumor (TURBT).

Exclusion criteria: Patients with incomplete clinical data; Patients with lymph node or distant metastasis; Patients receiving preoperative radiotherapy, chemotherapy, immunotherapy or targeted therapy; Patients with other malignant tumors; Patients with severe cardiovascular, liver and kidney, and blood system diseases; Immunocompromised persons.

### Methods

To those patients who underwent transurethral resection of bladder tumor (TURBT), Intravesical instillation of Epirubicin (50 mg) was performed regularly within 1 year after the operation; B-ultrasonography and cystoscopy were performed every 3 months after the operation. Complete the 5-year follow-up plan.

Histological grading using the WHO 2004 grading system; Pathological staging using the 2009 TNM staging system (UICC).

The content of this study has been approved by the Ethics Committee of our hospital.

### Reagents

Rabbit anti-human CA-IX polyclonal antibody, mouse anti-human Ki-67 monoclonal antibody, immunohistochemistry EnVision kit, and DAB chromogenic kit were purchased from Fuzhou Maixin Biotechnology Development Co., Ltd.

Take the archived wax block, make 3 serial sections of 4 μm thickness with a tissue microtome, 1 section is stained with HE, and the other 2 sections are attached to the glass slides pretreated with L-polylysine. The oxidase blocking solution was incubated at room temperature, soaked and rinsed in PBS, then placed in 0.01 mol/L citrate buffer (pH 6.0) for microwave repair, and cooled to room temperature. Blocked with 10% normal goat serum, incubated at room temperature, and added CA-IX and Ki-67 primary antibodies (1:100) overnight at 4 °C. Add biotin-labeled secondary antibody dropwise, and then dropwise add horseradish peroxidase-labeled streptavidin. DAB staining, hematoxylin counterstaining, and neutral gum mounting. Known positive sections were used as positive controls and phosphate-buffered saline (PBS) was used instead of primary antibodies as negative controls. Positive and negative control groups were set in each experiment. The experimental steps were strictly in accordance with the reagent instructions.

### Judgment of results

CA-IX is located in the cell membrane, and the corresponding position is light yellow to dark brown as the positive expression of the cell. Randomly select 10 high-power fields for each slice, count the total number of cells and the number of positive cells, and score according to the percentage of positive cells: < 10% is 0 points, 10%–50% is 1 point, 51%–75% is 2 points, 76%–100% is 3 points; according to the staining intensity score: 1 point for no staining, 2 points for light staining, 3 points for medium staining, and 4 points for dark staining. The product of the positive cell percentage score and the color intensity score is used as the judgment result: ≤ 3 is divided into no expression (-), 4 to 6 is divided into low expression, and 7 to 12 is divided into high expression. In the end, no expression or low expression is regarded as negative expression of CA-IX; high expression is regarded as positive expression of CA-IX. Two senior pathologists used a double-blind method to read the film.

### Statistical processing

SPSS 21.0 statistical software was used for statistical processing, the research data obeyed a normal distribution, and the *χ*^2^ test was used. *P* < 0.05 was considered statistically significant. Divided 194 bladder cancer patients into positive and negative groups according to the expression intensity of CA-IX; the correlation between the expression intensity of CA-IX and various parameters was analyzed. Univariate Logistic regression analysis was performed to find the risk factors of recurrence of bladder urothelial carcinoma after TURBT, furthermore, the factors obtained from univariate Logistic regression analysis were included in multivariate Logistic regression analysis to find the independent risk factors to predict recurrence of bladder urothelial carcinoma after TURBT (*P* < 0.05).The Kaplan–Meier survival curve was drawn based on the expression intensity of CA-IX, and the postoperative recurrence of the two groups was compared.

## Results

### Expression of CA-IX in bladder-related tissues

**Fig. 1:**
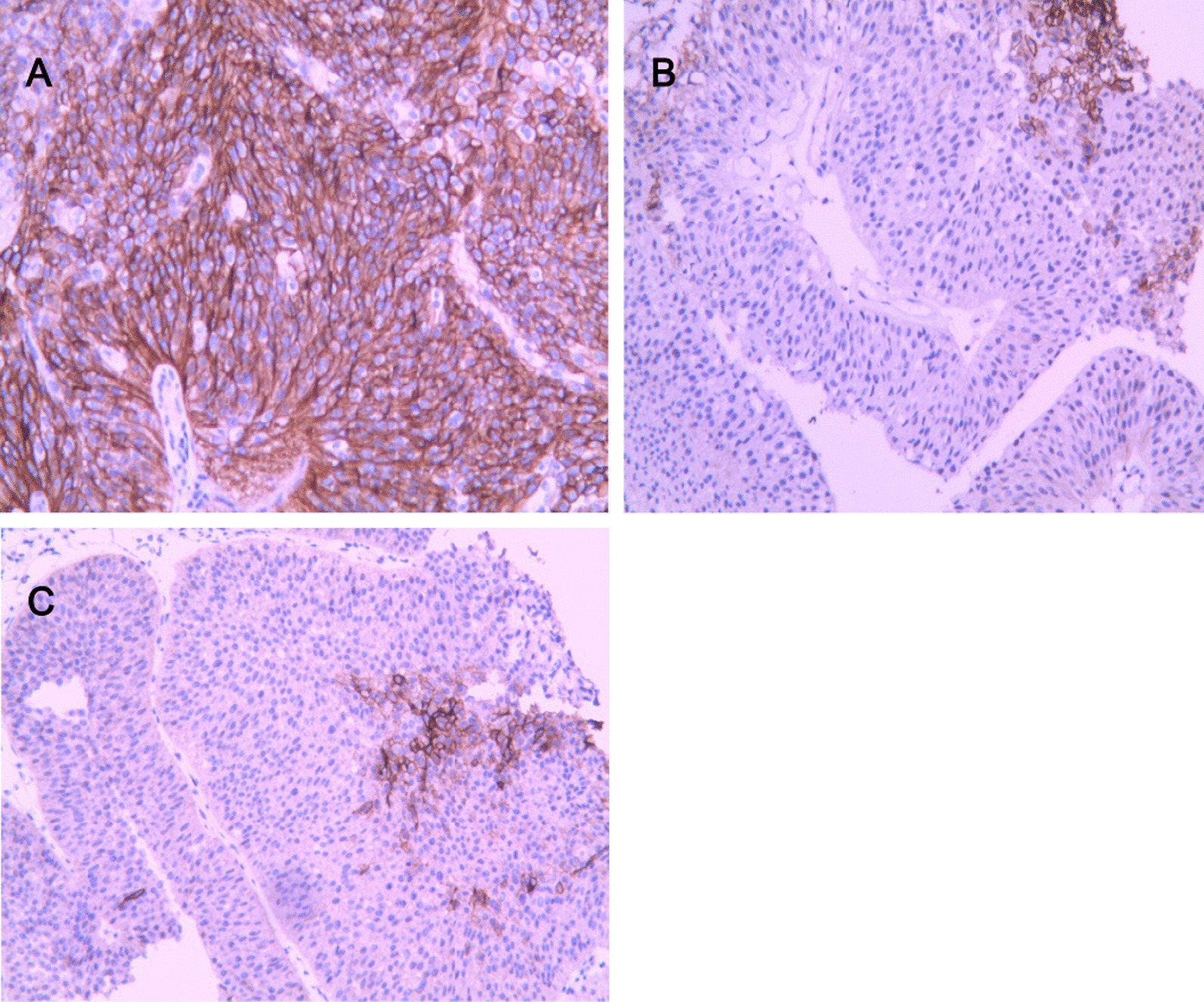
**A** CA-IX is diffusely expressed in bladder urothelial carcinoma, expressed on the cell membrane (SP method, × 100). **B** CA-IX is focally expressed in bladder urothelial adjacent tissues, which is cell membrane expression (SP method, × 100). **C** CA-IX is focally expressed in normal bladder tissue, expressed on the cell membrane (SP method, × 100)

Immunohistochemistry showed that CA-IX was expressed on the cell membrane, The positive rate of CA-IX in bladder urothelial carcinoma was 68.1% (132/194), the positive rate in adjacent tissues was 44.3% (86/194), and the positive rate in normal tissues was 27.3% (53/194). The expression of CA-IX in bladder urothelial carcinoma was significantly higher than that in adjacent tissues and normal tissues (*P* < 0.05). See Table 1 and Fig. 1A–C for details.

**Table 1:** Expression of CA-IX in bladder urothelial carcinoma tissues and other tissues

Group	CA-IX		*χ*^2^value	*P* value
	Positive	Negative		
Cancer tissue	132	62		
Paracancer tissue	86	108	22.15	0.00
Normal tissue	53	141	64.48	0.00

### General clinical information

During the study period, a total of 245 patients were enrolled, and all patients had their tumors resected successfully, and the specimens were confirmed by the pathology department. A total of 194 cases were followed up for 5 years, 42 cases were lost to follow-up, and 9 cases were incomplete information. There were 145 males and 49 females; 76 cases were < 60 years old, 118 cases were ≥ 60 years old, with an average age of 62.3 ± 12.2; 105 cases were < T1, 89 were T1; There were 80 cases of high differentiation and 114 cases of poor differentiation; 83 cases of single tumor and 111 cases of multiple tumors; 72 cases of tumor diameter ≥ 3 cm, 122 cases of tumor diameter < 3 cm; 76 cases had at least one recurrence during the 5-year follow-up period, and 118 cases had no recurrence; CA-IX expression was positive in 132 cases and negative in 62 cases. According to the expression of CA-IX, the research subjects were divided into positive group and negative group, and the age, gender, T stage, degree of differentiation, tumor number, tumor diameter, and recurrence status of the two observation groups were counted.

The expression of CA-IX had no statistical significance with age, gender and tumor diameter (*P* > 0.05); the expression of CA-IX had statistical significance with T stage, degree of differentiation, tumor number and recurrence (*P* < 0.05). See Table 2 for details.

**Table 2:** Clinically relevant parameters

Parameter	Number of cases	CA-IX	*χ*^2^ value	*P* value
		Positive (132)	Negative (62)	
Total	194			
Age			0.842	0.359
< 60 years old	76	55	21	
≥ 60 years old	118	77	41	
Gender			0.015	0.904
Male	145	99	46	
Female	49	33	16	
T stage			10.412	0.001
< T1	105	61	44	
T1	89	71	18	
Differentiation			5.405	0.02
Highly	80	47	33	
poorly	114	85	29	
Number of tumors			5.485	0.019
Single	83	64	19	
Multiple	111	68	43	
Tumor diameter			0.920	0.337
≥ 3 cm	72	52	20	
< 3 cm	122	80	42	
Recurrence			5.258	0.022
Yes	76	59	17	
No	118	73	45	

### Univariate Logistic regression analysis of bladder urothelial carcinoma recurrence

The study cases were divided into the recurrence group and the non-recurrence group according to the recurrence status, and the relevant clinical characteristics of the two study groups were summarized and analyzed. By incorporating the relevant parameters into the univariate Logistic regression analysis, we can draw the following conclusions.

Bladder urothelial carcinoma recurrence after TURBT had statistical significance (*P* < 0.05) with T stage (OR = 0.41, 95%Cl: 0.227–0.739, *P* = 0.003), degree of differentiation (OR = 0.511, 95%Cl: 0.279–0.935, *P* = 0.029), CA-IX expression (OR = 2.396, 95%Cl: 1.233–4.654, *P* = 0.01). Bladder urothelial carcinoma recurrence after TURBT had no statistical significance (*P* > 0.05) with age (OR = 0.895, 95%Cl: 0.496–1.613, *P* = 0.713), gender (OR = 1.227, 95%Cl: 0.636–2.369, *P* = 0.542), tumor number (OR = 0.556, 95%Cl: 0.307–1.010, *P* = 0.054) and tumor diameter (OR = 1.555, 95%Cl: 0.858–2.816, *P* = 0.145) (*P* > 0.05). (See Table 3 for details).

**Table 3:** Univariate logistic regression analysis of bladder urothelial carcinoma recurrence

Parameter	OR	95% confidence interval for EXP(B)		*P* value
		Lower limit	Upper limit	
Age	0.895	0.496	1.613	0.712
Gender	1.227	0.636	2.369	0.542
T stage	0.41	0.227	0.739	0.003
Differentiation	0.511	0.279	0.935	0.029
Number of tumors	0.556	0.307	1.010	0.054
Tumor diameter	1.555	0.858	2.816	0.145
CA-IX expression	2.396	1.233	4.654	0.01

### Multivariate Logisti regression analysis of bladder urothelial carcinoma recurrence

The risk factors were further included in the multivariate Logistic regression analysis according to the univariate Logistic regression analysis. After analysis, we obtained: clinical T stage of bladder urothelial carcinoma (OR = 0.446, 95%Cl: 0.241–0.826, *P* = 0.01), degree of differentiation (OR = 0.501, 95%Cl: 0.262–0.956, *P* = 0.036)),the number of tumors (OR = 0.502, 95%Cl: 0.266–0.947, *P* = 0.033) and CA-IX expression intensity (OR = 2.325, 95%Cl: 1.157–4.673, *P* = 0.018) were independent influencing factors for predicting the recurrence of bladder urothelial carcinoma after resection (*P* < 0.05); Tumor diameter (OR = 1.241, 95%Cl: 0.651–2.364, *P* = 0.512) was not statistically significant with recurrence after resection (*P* > 0.05). See Table 4 for details.

**Table 4:** Multivariate Logisti regression analysis of bladder urothelial carcinoma recurrence

Parameter	OR	95% confidence interval for EXP(B)		*P* value
		Lower limit	Upper limit	
T stage	0.446	0.241	0.826	0.01
Differentiation	0.501	0.262	0.956	0.036
Number of tumors	0.502	0.266	0.947	0.033
Tumor diameter	1.241	0.651	2.364	0.512
CA-IX expression	2.325	1.157	4.673	0.018

### Kaplan–Meier survival curve of CA-IX expression intensity and recurrence

This study enrolled 245 patients who underwent transurethral resection of bladder tumors in our hospital, and a total of 194 patients were followed up for 5 years. Urinary tract B-ultrasound and cystoscopy were evaluated every 3 months after surgery. Relapse status, time of relapse and clinical information of patients were recorded. During the follow-up period, 76 cases recurrence, 118 cases did not recured, and the recurrence rate was 39.17% (76/194). Among them, 59 cases relapsed in CA-IX positive expression group, the recurrence rate was 44.69% (59/132), and 17 cases relapsed in the negative expression group, and the recurrence rate was 27.41% (17/62). The mean recurrence time of CA-IX positive group was 29.93 ± 9.86 (months), and the mean recurrence time of CA-IX negative group was 34.02 ± 12.44 (months), *P* = 0.038 by *T* test of the two observation groups. The results showed that the recurrence rate and recurrence time of patients with positive expression of CA-IX in bladder urothelial carcinoma were significantly higher than those of patients with negative expression of CA-IX.

**Fig. 2:**
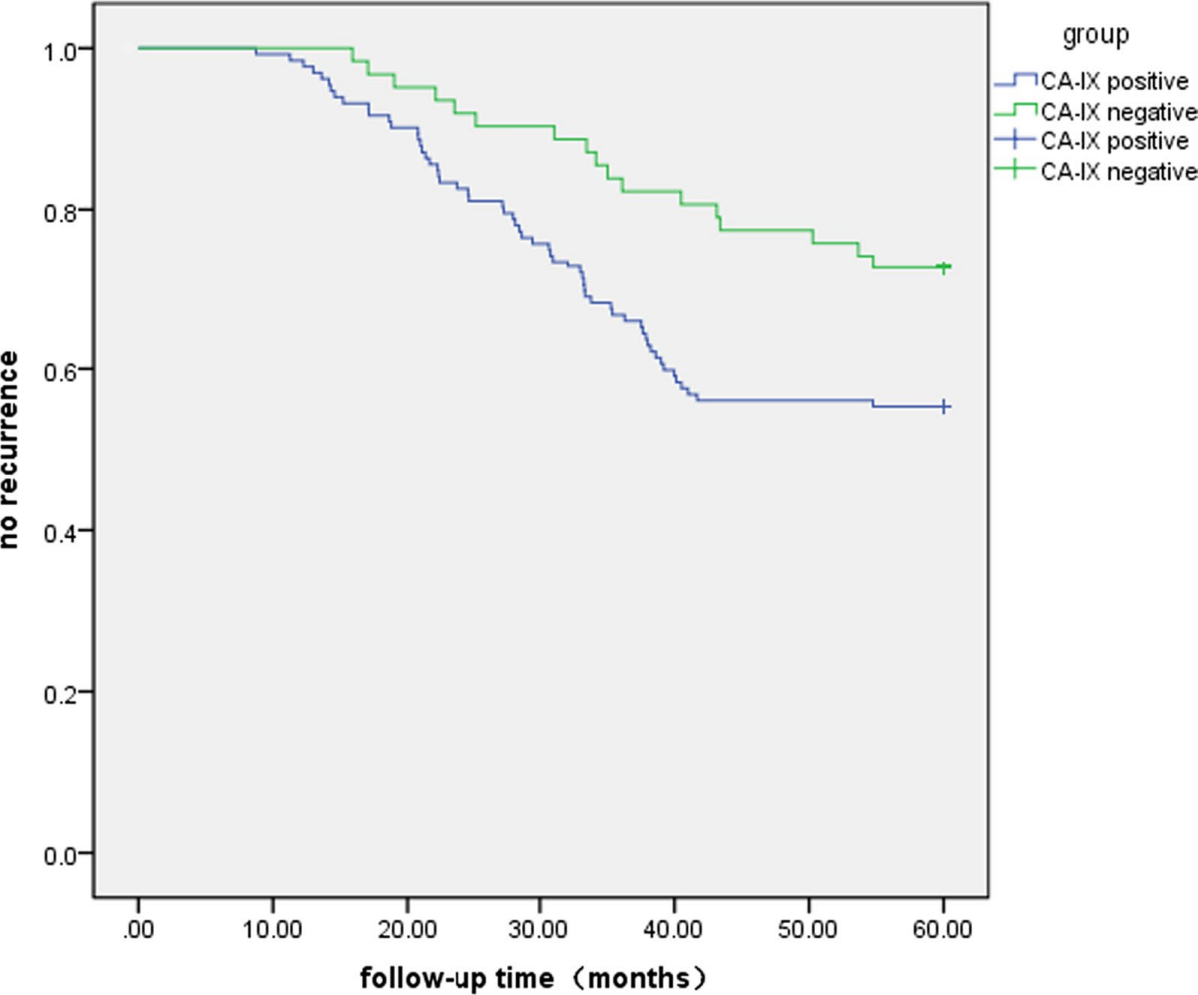
CA-IX expression intensity and survival curve of bladder urothelial carcinoma recurrence after resection (months)

According to the expression of CA-IX, the relapsed patients were divided two groups, and then the Kaplan–Meier survival curve was drawn. The curve showed that the recurrence rate of CA-IX positive expression group was significantly higher than that of negative group (*P* < 0.05). See Fig. 2 for details.

## Discussion

Bladder urothelial carcinoma is a common malignant tumor of the urinary system. Transurethral resection of bladder tumor and radical cystectomy is the main surgical methods. The physical damage caused by surgery and postoperative life quality of patients are greatly different between the two surgical methods. It is very important for patients to choose a reasonable surgical method. The surgical indication for transurethral resection of bladder tumor is the non-muscle invasive bladder cancer. Preoperative CT and cystoscopy are the main methods for staging assessment. However, the clinical staging predicted before surgery is often lower than the actual pathology staging in clinical work, Thus causing a considerable part of patients to experience recurrence or even disease progression after surgery [15]. Therefore, accurate preoperative clinical staging is crucial in the treatment of bladder urothelial carcinoma.

CA-IX is a transmembrane glycoprotein composed of acidic amino acids, and is a cell membrane expressed protein. CA-IX has a very low expression in normal tissues, but is abundantly expressed in various types of tumors, making cancer cells resistant to hypoxic environment, maintaining a relatively stable pH in cancer cells, and promoting the proliferation and differentiation of cancer cells [16]. At present, a large number of literatures have confirmed that CA-IX is significantly expressed in various malignant tumors, but it is negative in the normal group [17, 18]. Vats, L’s study showed that CA-IX was strongly expressed in renal clear cell carcinoma [19]; Ambrosio, MR’s study showed that CA-IX expression was positively correlated with prostate cancer grade and stage and outcome [20]; Supuran, CT [21] found that CA-IX was involved in the occurrence and migration of breast cancer. At present, CA-IX is recognized as a molecular marker of malignant tumors, and CA-IX has become a significant target in anti-cancer therapy, providing a new direction for targeted drugs [22].

This study retrospectively analyzed the clinical data of 194 patients diagnosed and treated in our hospital, and found that the positive rate of CA-IX in bladder urothelial carcinoma was 68.1% (132/194), and the positive rate in adjacent tissues was 44.3% (86/194), the positive rate of normal tissue was 27.3% (53/194). This revealed that CA-IX is significantly expressed in bladder urothelial carcinoma and is a meaningful tumor marker for bladder urothelial carcinoma, which may provide a new method for molecular diagnosis of bladder urothelial carcinoma [23].

The recurrence rate of bladder urothelial carcinoma after resection is high, and it is currently recognized that the degrees of differentiation, tumor number, tumor diameter, and pathological stage are risk factors for predicting the recurrence of bladder urothelial carcinoma [24, 25]. In this study, through Logistic univariate and multivariate analysis, it was found that the clinical T stage of bladder urothelial carcinoma (OR = 0.446, 95%Cl: 0.241–0.826, *P* = 0.01), the degree of differentiation (OR = 0.501, 95%Cl): 0.262–0.956, *P* = 0.036) and CA-IX expression intensity (OR = 2.325, 95%Cl: 1.157–4.673, *P* = 0.018) were independent influencing factors for predicting the recurrence of bladder urothelial carcinoma after resection. This indicates that CA-IX plays an important role in the occurrence and development of bladder urothelial carcinoma. This result also confirms the research of domestic and foreign scholars [26].

We conducted long-term follow-up of surgical patients. 245 patients were enrolled, and a total of 194 patients completed the 5-year follow-up, 42 patients were lost to follow-up, 9 patients had incomplete data, and 194 patients finally completed the follow-up plan. During the follow-up period, 76 cases recurred, 118 cases did not recur, and the recurrence rate was 39.17% (76/194). Among them, 59 cases relapsed in CA-IX positive expression group, the recurrence rate was 44.69% (59/132), and 17 cases relapsed in the negative expression group, and the recurrence rate was 27.41% (17/62). The mean recurrence time of CA-IX positive group was 29.93 ± 9.86 (months), and the mean recurrence time of CA-IX negative group was 34.02 ± 12.44 (months). It was found that the recurrence rate and recurrence time of patients with positive expression of CA-IX in bladder urothelial carcinoma were significantly higher than those of patients with negative expression of CA-IX (*P* < 0.05) by plotting the Kaplan–Meier survival curve. This phenomenon indicates that the expression of CA-IX is a meaningful tumor marker for predicting the recurrence of bladder urothelial carcinoma. Combined with the expression intensity of CA-IX before surgery, it can provide a reference for the selection of surgical methods and improve the therapeutic effect. Patients with positive CA-IX expression or a large number of tumors, poor differentiation, and advanced stages should be followed up closely after surgery to avoid missing recurrence or progression, which may lead to the progression of some patients to muscle-invasive bladder cancer, eventually having to undergo radical cystectomy. Our study shows that high CA-IX expression can lead to poor prognosis, positive expression is closely related to recurrence rate, CA-IX is an independent risk factor for predicting recurrence of bladder urothelial carcinoma after resection.

To sum up, It CA-IX is an excellent tumor marker for bladder urothelial carcinoma, but it is not a specific tumor marker of bladder, and the expression of CA-IX can be used to predict bladder urothelial carcinoma after resection. There is a certain reference value later. It CA-IX protein has a certain role in the occurrence and progression of bladder urothelial carcinoma, which is a topic worthy of further study, and can provide a new direction for the development of targeted drugs. However, the sample of this study is limited, the experimental design is a retrospective study, and the postoperative follow-up period is not long, and the final conclusion needs further research to confirm, In addition, the expression and effect of CA-IX in bladder muscle-invasive urothelial carcinoma is also worth studying.

## Data Availability

We cannot provide and share our datasets in publicly available repositories because of informed consent for participants as confidential patient data. Data may be obtained from the corresponding author upon reasonable request.
